# Effectiveness of pharmacological agents for the treatment of non-infectious scleritis: a systematic review protocol

**DOI:** 10.1186/s13643-020-01314-9

**Published:** 2020-03-12

**Authors:** Sathana Ingaralingam, Saaeha Rauz, Philip I. Murray, Robert J. Barry

**Affiliations:** 1 Academic Unit of Ophthalmology, Institute of Inflammation and Ageing, University of Birmingham, Birmingham and Midland Eye Centre, City Hospital, Dudley Road, Birmingham, B18 7QU UK; 2 Institute of Clinical Sciences, College of Medical and Dental Sciences, University of Birmingham, Edgbaston, Birmingham, B15 2TT UK

**Keywords:** Systematic review, Non-infectious scleritis, Management, Pharmacological agent, Drug therapy, Meta-analysis

## Abstract

**Background:**

Non-infectious scleritis is a potentially sight-threatening condition in which the sclera, the white outer layer of the eye, becomes inflamed. Whilst scleritis can be infective, the majority of cases are due to non-infectious causes, often occurring in association with an underlying systemic autoimmune or auto-inflammatory condition. Thorough systemic work-up is crucial to identify disease aetiology and exclude infection; however, a significant proportion of disease remains idiopathic with the underlying cause unknown. Non-infectious scleritis is normally managed with systemic corticosteroid and immunosuppression, yet there is no widely agreed consensus on the most appropriate therapy, and no national or international guidelines exist for treatment of non-infectious scleritis.

**Methods:**

Standard systematic review methodology will be used to identify, select and extract data from comparative studies of pharmacological interventions used to treat patients with non-infectious scleritis. Searches of bibliographic databases (Cochrane Library, MEDLINE, CINAHL and EMBASE) and clinical trial registers will be employed. No restrictions will be placed on language or date of publication. Non-English articles will be translated where necessary. The primary outcome of interest will be disease activity measured by reduction in scleritis grading according to standardised grading systems. Secondary outcomes will include change in best corrected visual acuity, reduction in concurrent dose of systemic corticosteroid, time to treatment failure, adverse events and health-related quality of life. Risk of bias assessment will be conducted appropriate to each study design. Study selection, data extraction and risk of bias assessment will be completed by two reviewers independently. Data will be presented in a table and a narrative synthesis will be undertaken. Meta-analysis will be performed where methodological and clinical homogeneity exists. Subgroup and sensitivity analysis will be undertaken if appropriate.

**Discussion:**

Many studies have investigated the effectiveness of pharmacological agents used in the management of non-infectious scleritis. A systematic review is needed to collate and analyse this evidence. Findings of this systematic review will help guide ophthalmologists managing patients with non-infectious scleritis and may form the basis for evidence-based recommendations for future clinical practice and encourage standardisation of treatment protocols.

**Systematic review registration:**

PROSPERO CRD42019125198

## Background

Non-infectious scleritis is a potentially sight-threatening condition in which the sclera becomes inflamed and oedematous. It is usually characterised by severe pain that is often worse at night, severe enough to wake patients from sleep and pain on ocular movement. The globe is typically very tender to palpation [1]. In anterior scleritis, the eye is red, although this may not be visibly present in isolated posterior scleritis. Other symptoms include photophobia if there is corneal involvement. These symptoms may be so severe that they limit activities of daily living [2]. Non-infectious scleritis is more commonly seen in females and typically peaks in the fourth to fifth decade of life. The prevalence is approximately 6 per 10,000 in the US population [3]. Non-infectious scleritis is associated with significant ocular comorbidity and reduced quality of life [4]. Complications may occur due to the disease process or treatment of disease and include keratitis, cataract formation, optic disc swelling, uveitis and corneal and scleral thinning that can result in globe perforation [5]. Approximately 40 to 50% of patients with non-infectious scleritis have an underlying systemic autoimmune condition, such as rheumatoid arthritis, granulomatosis with polyangiitis, microscopic polyangiitis, relapsing polychondritis, systemic lupus erythematous and seronegative spondylarthropathies [4, 6]. Infectious scleritis accounts for less than 10% of all cases and will not be discussed further [7].

Non-infectious scleritis is typically classified according to a grading system proposed by Watson and Hayreh in 1976 [5]; disease is classified according to the anatomical location of inflammation and is further subdivided by clinical features [4]. Scleritis is defined as anterior if the affected sclera is visible to the naked eye of an observer, or posterior if the affected sclera is enclosed by orbital tissues and therefore not visible to an observer [8]. Anterior scleritis is more common, accounting for up to 90% of cases. Anterior scleritis can be further categorised by clinical phenotype into diffuse, nodular and necrotizing types [5]. Diffuse anterior scleritis is typically the most ‘benign’ form and presents with dilation of deep episcleral vessels and areas of extensive of scleral oedema [5, 9]. Nodular scleritis often presents with multiple, well-defined nodules that are tender on palpation [10]. Although necrotizing scleritis is the least common form, it has been reported to be more strongly associated with systemic disease, is often more aggressive in clinical presentation [11] and may lead to areas of scleral thinning and ectasia with exposure of the underlying choroid (scleromalacia perforans) [1].

Diagnosis of posterior scleritis is often delayed as its clinical features may be confused with those of intraocular inflammation, acute orbital inflammation and ocular tumours [3, 12]. Posterior scleritis is usually unilateral, and patients often complain of a dull ache originating from behind the eye which may be worse with eye movement. Proptosis may also occur due to inflammation of peri-ocular tissues. Vision may be reduced due to associated macular oedema, serous retinal detachment and optic nerve head oedema. The eye is typically white. B-mode ultrasonography may show diffuse scleral thickening and accumulation of fluid around the optic nerve in the sub-tenon space demonstrating the pathognomonic T-sign [13]. Posterior scleritis is associated with a poorer visual prognosis than anterior scleritis [14].

Non-infectious scleritis is normally treated with systemic corticosteroid and often the addition of immunosuppressive agents. Recent retrospective data suggest scleritis remission occurs in a majority of patients by 3.1 years [15]. Non-necrotizing, non-infectious scleritis may be first managed with a non-steroidal anti-inflammatory drug (NSAID), such as flurbiprofen [16] or indomethacin [5]. Whilst NSAIDs have been shown to be effective in patients with a low degree of scleral inflammation [17], a significant proportion of patients eventually requires more potent immunosuppression in the form of corticosteroids. Although effective in rapidly reducing ocular inflammation, corticosteroids are associated with significant local and systemic side effects that are dose-dependent [18]. It is therefore necessary to minimize the dose and duration of corticosteroid therapy; corticosteroid-sparing agents are often introduced in patients with more chronic disease to enable tapering of the corticosteroid dose in order to balance the benefit-to-harm ratio [18]. Such corticosteroid-sparing immunosuppressive agents include methotrexate, mycophenolate mofetil and azathioprine. Cyclophosphamide, anti-tumour necrosis factor (TNF) or biologic therapies, including rituximab, may also be used for particularly severe or recalcitrant disease [19]. Pre-treatment checks and close monitoring of parameters, such as full blood count, renal and liver function is often necessary as these agents are associated with potentially serious side effects [20, 21].

Although many pharmacological agents have shown promising results in the management of non-infectious scleritis, there are currently no widely accepted management guidelines. Inevitably, this absence of guidance culminates in uncertainty for patients, clinicians and healthcare providers. An initial scoping search revealed several narrative reviews on the management of scleritis; however, no systematic reviews were identified. The most recent review, from 2013, summarised existing evidence on pharmacological management of non-infectious scleritis. It did not however, follow recognised systematic review methodology as it did not describe a clear prospective search strategy that may have resulted in the omission of relevant articles [4]. Furthermore, concerns arise over the lack of transparency in reporting as the review did not follow the Preferred Reporting Items for Systematic Reviews and Meta-Analyses (PRISMA) guidelines. Additionally, as the incidence and prevalence of scleritis are relatively low, there have been very few studies, particularly randomised controlled trials (RCTs), comparing treatments in large cohorts of patients.

Therefore, a systematic review of existing literature is necessary to evaluate and summarise the available evidence for the effectiveness of the many pharmacological agents in the treatment of non-infectious scleritis. This may form the basis of evidence-based recommendations for future clinical practice.

## Methods

### Aim

The aim of this study is to assess the effectiveness of the available pharmacological treatments in the treatment of scleritis. This will be achieved by conducting a systematic review of studies:

- Comparing a pharmacological agent to the non-use of a pharmacological agent
- Comparing a pharmacological agent to the same or another pharmacological agent

The protocol for this systematic review was registered with PROSPERO database (reference CRD42019125198) [22]. The review and its findings are reported in accordance with the PRISMA guidelines [23]. A PRISMA-P checklist for this protocol is shown in Additional File 1.

### Searches

The following sources will be searched for evidence to review:

- EMBASE
- MEDLINE, MEDLINE in process (Ovid)
- CINAHL
- The Cochrane Library (CENTRAL Register of Controlled Trials)

Registers of clinical trials:

- WHO International Clinical Trials Registry Platform (ICTRP) portal (www.who.int/ictrp)
- Clinicaltrials.gov (www.clinicaltrials.gov)
- European Clinical Trials Database (EudraCT)
- International Standard Randomised Controlled Trials Number Database (ISRCTN)
- UK Clinical Research Network (www.ukcrn.org.uk)

Abstract and conference proceedings:

- Conference Proceedings Citation Index (Web of Science)
- British library ZETOC

Dissertations, theses and grey literature:

- British library Ethos
- ProQuest (www.proquest.com)
- OpenGrey (www.opengrey.eu)

For bibliographic databases, the search strategy will combine index and free text terns for the pharmacological agents as a class (such as anti-TNF) and as individual drugs (such as etanercept and adalimumab) and the condition. Searches will also be made for specific diagnoses where scleritis is a common feature; this includes rheumatoid arthritis, granulomatosis with polyangitis, relapsing polychondritis, systemic lupus erythematous, Behçet’s disease and sarcoidosis.

A sample strategy from MEDLINE has been included as Appendix 1; this will be adapted for each of the databases above. These sources will be searched in an iterative manner. In order to ensure no relevant primary studies are missed, the bibliographies of relevant systematic reviews will be hand-searched. Additionally, a clinical expert in the field will be contacted to ensure there are no other similar ongoing systematic reviews. No restrictions will be placed on either language or date of publication. Search results will be managed on an EndNote database; this will aid the removal of duplicate entries, study details and references. Restricting the search to electronic databases only could introduce publication bias; this will be limited by searching grey literature.

### Selection criteria

The following criteria will be utilised to select studies for this review:

- Study design
  - ○ RCTs and other comparative studies (non-randomised controlled trials, comparative observational studies)

### Participants

- Participants of any age, gender or ethnicity with non-infectious scleritis

- Intervention and comparator
  - ○ Comparing any pharmacological agent administered via any route, to no use of a pharmacological agent.
  - Comparing any pharmacological agent administered via any route, to the same or another pharmacological agent administered via any route.
- Outcomes
  - ○ Primary outcome
    - ■ Disease activity (measured by reduction in scleritis grading)
  - ○ Secondary outcomes
    - ■ Reduction in concurrent dose of systemic corticosteroid
    - ■ Time to treatment failure
    - ■ Change in best corrected visual acuity
    - ■ Adverse events
    - ■ Health-related quality of life

Non-infectious scleritis will be defined as cases of scleritis where no infective organism has been identified. All types and severity non-infectious scleritis will be eligible for inclusion; this will include both anterior (diffuse, nodular and necrotizing) and posterior non-infectious scleritis. We recognise that there may be instances where both infectious and non-infectious scleritis are included in a trial. In this instance, studies will only be included in the review if there is a subgroup analysis enabling identification and examination of non-infectious cases.

Outcome data will be collected for all reported time-points, before categorised as follows for further analysis: ≤ 3 months, > 3 and ≤ 6 months and > 6 months. The > 6 months category may be further subdivided if long-term data is available. Treatment failure will be defined as the failure to achieve the primary treatment outcome as specified by each author. This could include non-response (i.e. no improvement), a worsening of inflammation (as assessed by the author) or the need for additional/rescue therapy.

### Selection Process

The study selection process comprises two stages:

1. Title and abstract of the articles from the search will be screened in order to remove irrelevant records.
2. The full text of potentially relevant articles will be retrieved and assessed against the selection criteria.

Two reviewers (SI and RJB) will independently assess the suitability of articles; any differences in opinion will be resolved by discussion, and if needed, referral to a third reviewer (PIM). The selection process will be piloted and if necessary, modified. The process will be outlined using a PRISMA flow diagram [23]. Details and reasons for excluding articles in the second stage will be recorded. Where possible, any non-English language articles will be translated in order to aid study selection and analysis.

### Data extraction

Two reviewers will independently extract relevant data from the suitable articles. Any disagreements will be resolved by discussion, and if required, referral to a third colleague. A standardised piloted data extraction form will be used to collate the data. If further information is required, study authors and publishing bodies may be contacted. For each article, the following information, but not limited to, will be extracted:

- Study characteristics
  - ○ Authors, publication year, title and journal
  - ○ Study design
  - ○ Setting
  - ○ Sample size
  - ○ Length of follow-up
  - ○ Analysis
- Participant characteristics
  - ○ Patient selection and recruitment criteria
  - ○ Demographic data number, age, gender, socioeconomic status and ethnicity
  - ○ Type of scleritis (defined by anatomical location, pattern of inflammation and aetiology if reported)
  - ○ Comorbidity
  - ○ Concurrent medication
- Intervention and comparator
  - ○ Pharmacological agents studied and regimen (dose, frequency, route of administration)
  - ○ Comparator details (where present)
  - ○ Any differences in underlying care between treatment groups
- Outcomes and finding
  - ○ Outcomes being measured and results for each outcome
  - ○ Precision and statistics test results for each outcome
  - ○ Completeness of follow-up for each outcome

### Quality assessment

Two reviewers will independently undertake quality assessment of all included articles (SI and RJB). Any differences will be resolved by discussion and if necessary, an opinion from a third reviewer (PIM) will be sought. The Cochrane Handbook risk of bias tool will be used to assess RCTs [24]. Non-randomised trials will also be assessed using this tool, but it is acknowledged that the criteria for randomisation and allocation concealment will not be relevant. Guidelines outlined in chapter 13 of the Cochrane Handbook will be used to assess prospective controlled observational studies [24]; however, the risk of bias tool for RCTs can also be adapted and used as a minimum assessment, again accepting that not all criteria will be relevant. In these studies, the most relevant criteria to evaluate is how groups were selected, differences in patient characteristics, loss to follow-up, biases and confounding in outcome assessment. Case-controlled studies will be assessed using the Newcastle-Ottawa scale [25]. A summary of the assessment of bias of individual studies will be included in the findings’ table.

### Analysis

Studies will be grouped by intervention and comparator. Data will be presented in a table and a narrative synthesis of evidence conducted for each outcome of relevance to the review. This will provide a summary of the findings from each study and identify patterns in the data. Clinical and methodological heterogeneity will be assessed for each comparison and outcome. This will determine the feasibility for undertaking a meta-analysis and whether a random effects or fixed effect model is most appropriate [26]. The *I*^2^ statistic (percentage of total variability in data due to study heterogeneity) and tau-squared statistic (represents the extent of variation among the effects observed in different studies) will be reported where appropriate. Data from studies with differing study designs will not be pooled together. A forest plot may be produced to show the pooled effect of findings. The possibility of publication bias will be investigated, and a funnel plot will be generated for each meta-analysis containing 10 or more studies [27]. It is expected that there will be multiple time-point data within each study itself, and between studies; data will be categorised for analysis into the following groups’ post interventions: ≤ 3 months, > 3 and ≤ 6 months and > 6 months. The > 6 months category may be subdivided if long-term data is available.

Results for study outcomes may be presented using a number of different measures within the same study and between studies. Scleritis activity may be measured by a clinical grading scale; changes in activity may be reported as a reduction or increase in grade, or as a proportion of patients achieving a pre-determined threshold (for example, a two-step improvement in activity grading). It might be necessary to convert data between formats in order to maximise data available for each analysis. For example, visual acuity can be reported as distance from Snellen charts, number of lines read from ETDRS charts, a LogMAR score or as the change in acuity. It may be possible to convert data from between these different formats. Any conversion of data will be performed with caution and regard to known issues; the use of converted data will be explicitly stated. Where multiple studies report comparable continuous data, such as health-related quality of life, using the same scale, data will be pooled using mean difference. Data derived from different scales may be pooled to generate a standardised mean difference.

If there is sufficient data available, a subgroup analysis may be undertaken if appropriate. This will be performed by grouping the data into categories, which may influence the outcome. For example, this might be clinical (e.g. route of administration) or anatomical (anterior vs posterior) classification of scleritis. Similarly, sensitivity analysis, to determine the robustness of the observed outcomes, may be conducted if a meta-analysis is undertaken. This could involve conducting a repeat of the primary analysis and substituting studies in the meta-analysis to observe the impact on the overall effect. Issues suitable for sensitivity analysis will only be identified during the review process [24].

### Reporting

This systematic review and its findings will be reported in accordance with the PRISMA guidelines [23]. The strengths and weaknesses of the review methodology and existing evidence will be discussed with regard to the external and internal validity of the findings. The potential implications of the review findings on current and future clinical practice will be explored. This may also highlight areas for possible future research.

### Discussion and potential impact

Non-infectious scleritis is a potentially blinding ocular inflammatory disease. However, there is wide variation in treatment reflecting limitations in primary data and a lack of national guideline or consensus statement. This review will systematically and comprehensively retrieve published evidence from a wide range of sources to evaluate the pharmacological treatment of non-infectious scleritis. Furthermore, this review will provide valuable information regarding the effectiveness of pharmacological agents compared with other active agents or placebo. This protocol is the first of its kind to be published, and the first to be registered prospectively.

This review will provide a clear reference point for UK/international specialists and should help to increase standardisation of clinical practice in line with current evidence, improve outcomes for patients and help to minimize harm from inappropriate therapies.

### Supplementary information

**Additional file 1.** PRISMA-P Checklist: Recommended items to include in a systematic review protocol.

## Data Availability

Not applicable

## Appendix

**Table 1:** MEDLINE sample search strategy

Search number	Search details
1	Exp Scleritis/
2	(Scleritis OR Scleritic) ti, ab.
3	1 OR 2
4	(GPA OR granulomatosis with polyangitis OR Wegener’s granulomatosis or WG) ti, ab.
5	(RA OR rheumatoid arthritis) ti, ab.
6	Relapsing Polychondritis ti, ab.
7	(Systemic Lupus Erythematosus OR SLE) ti, ab.
8	Sarcoidosis. ti, ab.
9	(Behçet’s Disease OR BD) ti, ab.
10	Therap*. ti, ab.
11	Treat*. ti, ab.
12	Management. ti, ab.
13	Drug. ti, ab.
14	Agent. ti, ab.
15	Corticosteroid*. ti, ab.
16	Prednisolone. ti, ab.
17	Prednisone. ti, ab.
18	(Cyclosporin OR ciclosporin) ti, ab.
19	Tacrolimus. ti, ab.
20	Voclosporin. ti, ab.
21	Sirolimus. ti, ab.
22	Azathioprine. ti, ab.
23	Methotrexate. ti, ab.
24	Mycophenolate mofetil. ti, ab.
25	Chlorambucil. ti, ab.
26	Cyclophosphamide. ti, ab.
27	Anti-TNF. ti, ab.
28	Adalimumab. ti, ab.
29	Certolizumab. ti, ab.
30	Golimumab. ti, ab.
31	Infliximab. ti, ab.
32	Etanercept. ti, ab.
33	Efalizumab. ti, ab.
34	Rituximab. ti, ab.
35	Abatacept. ti, ab.
36	Alemtuzumab. ti, ab.
37	Anakinra. ti, ab.
38	Canakinumab. ti, ab.
39	Gevokizumab. ti, ab.
40	Daclizumab. ti, ab.
41	Tocilizumab. ti, ab.
42	Secukinumab. ti, ab.
43	Interferon. ti, ab.
44	Fingolimod. ti, ab.
45	Aflibercept. ti, ab.
46	(Intravenous Immunoglobulin OR IVIG) ti, ab.
47	Colchicine. ti, ab.
48	Rilonacept. ti, ab.
49	Gevokizumab. ti, ab.
50	Apremilast. ti, ab.
51	Sulfasalazine. ti, ab.
52	Azithromycin. ti, ab.
53	Rebamipide. ti, ab.
54	(Non-Steroidal Anti-inflammatory Drug OR NSAID) ti, ab.
55	Ibuprofen. ti, ab.
56	Indomethacin. ti, ab.
57	Naproxen. ti, ab.
58	Celecoxib. ti, ab.
59	Triamcinolone. ti, ab.
60	Everolimus. ti, ab.
61	4 OR 5 OR 6 OR 7 OR 8 OR 9 OR 10 OR 11 OR 12 OR 13 OR 14 OR 15 OR 16 OR 17 OR 18 OR 19 OR 20 OR 21 OR 22 OR 23 OR 24 OR 25 OR 26 OR 27 OR 28 OR 29 OR 30 OR 31 OR 32 OR 33 OR 34 OR 35 OR 36 OR 37 OR 38 OR 39 OR 40 OR 41 OR 42 OR 43 OR 44 OR 45 OR 46 OR 47 OR 48 OR 49 OR 50 OR 51 OR 52 OR 53 OR 54 OR 55 OR 56 OR 57 OR 58 OR 59 OR 60
62	3 AND 61

## Abbreviations

CENTRAL: Cochrane Central Register of Controlled Trials
CINAHL: Cumulative Index to Nursing and Allied Health Literature
ETDRS: Early Treatment of Diabetic Retinopathy Study
MEDLINE: Medical Literature Analysis and Retrieval System Online
NSAID: Non-steroidal anti-inflammatory drug
PRISMA: Preferred Reporting Items for Systematic Reviews and Meta-Analyses
RCT: Randomised controlled trial
TNF: Tumour necrosis factor
