# Supplementary material for: Effectiveness of pharmacological agents for the treatment of non-infectious scleritis: a systematic review protocol
Source: Syst Rev. 2020 Mar 12;9:54. doi: 10.1186/s13643-020-01314-9 (PMC7068966; doi:10.1186/s13643-020-01314-9)
Supplement: Supplementary file 1 — Additional file 1. PRISMA-P Checklist: Recommended items to include in a systematic review protocol. [file 13643_2020_1314_MOESM1_ESM.docx]

**Additional File 1: PRISMA-P Checklist**

| **PRISMA-P 2015 checklist: recommended items to include in a systematic review protocol^a^** | | | |
| --- | --- | --- | --- |
| **Section/topic** | **Item #** | **Page #** | **Checklist item** |
|  |  |  | **ADMINISTRATIVE INFORMATION** |
|  |  |  | **Title** |
| **Identification** | 1a | 1 | Identify the report as a protocol of a systematic review |
| **Update** | 1b |  | If the protocol is for an update of a previous systematic review, identify as such |
| **Registration** | 2 | 3 | If registered, provide the name of the registry (e.g., PROSPERO) and registration number |
|  |  |  | **Authors** |
| **Contact** | 3a | 1 | Provide name, institutional affiliation, and e-mail address of all protocol authors; provide physical mailing address of corresponding author |
| **Contributions** | 3b | 19 | Describe contributions of protocol authors and identify the guarantor of the review |
| **Amendments** | 4 |  | If the protocol represents an amendment of a previously completed or published protocol, identify as such and list changes; otherwise, state plan for documenting important protocol amendments |
|  |  |  | **Support** |
| **Sources** | 5a | 19 | Indicate sources of financial or other support for the review |
| **Sponsor** | 5b | 19 | Provide name for the review funder and/or sponsor |
| **Role of sponsor/funder** | 5c | 19 | Describe roles of funder(s), sponsor(s), and/or institution(s), if any, in developing the protocol |
|  |  |  | **INTRODUCTION** |
| **Rationale** | 6 | 4-7 | Describe the rationale for the review in the context of what is already known |
| **Objectives** | 7 | 8 | Provide an explicit statement of the question(s) the review will address with reference to participants, interventions, comparators, and outcomes (PICO) |
|  |  |  | **METHODS** |
| **Eligibility criteria** | 8 | 10-11 | Specify the study characteristics (e.g., PICO, study design, setting, time frame) and report characteristics (e.g., years considered, language, publication status) to be used as criteria for eligibility for the review |
| **Information sources** | 9 | 8-9 | Describe all intended information sources (e.g., electronic databases, contact with study authors, trial registers, or other grey literature sources) with planned dates of coverage |
| **Search strategy** | 10 | 23-24 | Present draft of search strategy to be used for at least one electronic database, including planned limits, such that it could be repeated |
|  |  |  | **Study records** |
| **Data management** | 11a | 12-14 | Describe the mechanism(s) that will be used to manage records and data throughout the review |
| **Selection process** | 11b | 12 | State the process that will be used for selecting studies (e.g., two independent reviewers) through each phase of the review (i.e., screening, eligibility, and inclusion in meta-analysis) |
| **Data collection process** | 11c | 12-14 | Describe planned method of extracting data from reports (e.g., piloting forms, done independently, in duplicate), any processes for obtaining and confirming data from investigators |
| **Data items** | 12 | 12-14 | List and define all variables for which data will be sought (e.g., PICO items, funding sources), any pre-planned data assumptions and simplifications |
| **Outcomes and prioritization** | 13 | 12-14 | List and define all outcomes for which data will be sought, including prioritization of main and additional outcomes, with rationale |
| **Risk of bias in individual studies** | 14 | 14 | Describe anticipated methods for assessing risk of bias of individual studies, including whether this will be done at the outcome or study level, or both; state how this information will be used in data synthesis |
|  |  |  | **Data** |
| **Synthesis** | 15a | 14-16 | Describe criteria under which study data will be quantitatively synthesized |
|  | 15b | 14-16 | If data are appropriate for quantitative synthesis, describe planned summary measures, methods of handling data, and methods of combining data from studies, including any planned exploration of consistency (e.g., *I*^2^, Kendall’s tau) |
|  | 15c | 14-16 | Describe any proposed additional analyses (e.g., sensitivity or subgroup analyses, meta-regression) |
|  | 15d | 14-16 | If quantitative synthesis is not appropriate, describe the type of summary planned |
| **Meta-bias(es)** | 16 | 14-16 | Specify any planned assessment of meta-bias(es) (e.g., publication bias across studies, selective reporting within studies) |
| **Confidence in cumulative evidence** | 17 | 14-16 | Describe how the strength of the body of evidence will be assessed (e.g., GRADE) |
